# Symptom expression and coping strategies in women-focused attention-deficit/hyperactivity disorder communities: a comparative natural language analysis of Reddit

**DOI:** 10.3389/fpsyt.2026.1867179

**Published:** 2026-08-07

**Authors:** Joohee Seo, Youngsoo Kim, Sohyeon Ryu, Sunjoong Kim, Lidia Zylowska

**Affiliations:** 1 Department of Neuropsychiatry of Korean Medicine, National Medical Center, Seoul, Republic of Korea; 2 Integro Medi Lab Co., Ltd., Seoul, Republic of Korea; 3 Department of Internal Medicine of Korean Medicine, National Medical Center, Seoul, Republic of Korea; 4 Department of Clinical Korean Medicine, Graduate School, Kyung Hee University, Seoul, Republic of Korea; 5 Department of Psychiatry, University of Minnesota Medical School, Minneapolis, MN, United States

**Keywords:** ADHD, coping strategies, natural language processing, Reddit, social media analysis, women-focused ADHD communities

## Abstract

**Introduction:**

Attention-deficit/hyperactivity disorder (ADHD) in women remains underrecognized despite increasing evidence of gender-specific symptom patterns and treatment experiences. Using computational text analysis, this study examined how ADHD is expressed and managed in two Reddit communities: r/adhdwomen and r/ADHD.

**Methods:**

We collected and analyzed 3,383 unique, publicly available posts (r/adhdwomen: n = 1,414; r/ADHD: n = 1,969) using GPT-assisted natural language processing and statistical comparison methods. To ensure consistent output while identifying patterns of symptom expression and coping strategies, we set the model’s randomness parameter to 0.2 and ran it using Python 3.11.

**Results:**

Both groups frequently discussed core DSM-5 symptoms (inattention, hyperactivity/impulsivity). However, posts in r/adhdwomen contained more references to emotional dysregulation and internalizing symptoms (53.0% vs 47.3%, false discovery rate [FDR]-adjusted *p* = 0.025) and hormonal and life cycle influences (3.6% vs 0.4%, FDR-adjusted *p* < 0.001). The overall frequencies of the coping strategy subcategories did not differ significantly between the groups (all FDR-adjusted *p* > 0.05). The use of medication was the most frequently mentioned coping strategy followed by executive function support. Additionally, pairwise association analysis identified several recurrent coping combinations within each community. Across both subreddits, significant associations were consistently observed for executive function support combined with environmental structuring, digital tools, and social support. In r/adhdwomen, unique associations of psychotherapy with social support and mindfulness and emotion regulation with lifestyle emerged.

**Conclusion:**

The women-focused ADHD community (r/adhdwomen) more frequently discussed emotional dysregulation and internalizing symptoms as well as hormonal and life cycle influences than the general ADHD community (r/ADHD). Although the two communities showed similar overall frequencies of coping strategy categories, they partly differed in the recurring combinations of strategies discussed within posts. These findings should be interpreted as community-level discourse patterns rather than direct differences between women and men with ADHD because r/ADHD is a mixed-gender general ADHD community rather than a male comparison group. Social media text can be used as a supplementary source for understanding how ADHD-related experiences and coping strategies are discussed in everyday peer contexts.

## 1 Introduction

Attention-deficit/hyperactivity disorder (ADHD) is a neurodevelopmental disorder characterized by a pervasive pattern of inattention, hyperactivity, and impulsivity (1). Although typically diagnosed in childhood, ADHD often persists into adulthood, with a global rate of approximately 2.5% to 5% in adults (2, 3). Sex and gender differences in prevalence show that although boys are seven to eight times more likely to receive a childhood diagnosis than girls, women and men are diagnosed at nearly equal rates in adulthood (4). This pattern was previously interpreted as indicating higher male risk (5); however, emerging work suggests that ADHD may be underrecognized in girls and women (4, 6). Historically, ADHD has been associated with hyperactive boys, contributing to diagnostic bias and missed identification in girls and women (7, 8).

Overall, women with ADHD tend to present with the inattentive subtype and fewer overt behavioral disturbances such as conduct disorders (5), often leading to delayed diagnosis and intervention (9). Social and gender roles can also introduce expectations unique to women, including responsibilities such as household management and caregiving, which may interact with ADHD symptomatology. This can contribute to camouflaging or compensatory behaviors that hide ADHD-related difficulties and may delay recognition and diagnosis (8, 10). Although some of these coping strategies may work in the short term, they can lead to long-term fatigue, emotional distress, and identity fragmentation. Consequently, many women with ADHD initially present clinically through comorbid paths, including anxiety and depression (11), complicating accurate diagnosis and treatment. Furthermore, recent research suggests that ADHD in women is associated with specific psychosocial and physiological processes. Hormonal fluctuations linked to the menstrual cycle, pregnancy, and menopause have been reported to modulate ADHD symptom severity and treatment responsiveness (12, 13). Recent reviews and meta-analyses (7, 14, 15) indicate that ADHD symptoms in women may present a different profile than in men. Although gender and sex differences in ADHD symptoms are increasingly recognized, these differences remain underexplored in ADHD. The persistent research gap in understanding women’s ADHD experiences is likely related to the underrepresentation of women in ADHD samples and the limited ability of structured clinical data to capture subjective realities (16).

Beyond clinical and research settings, social media has become a widely used space in which people disclose mental health concerns, seek support, and participate in peer communities (17). Computational text analysis of content on platforms such as Twitter/X, Reddit, and TikTok has been applied across mental health contexts to characterize symptom language, public perceptions, risk signals, and treatment-related discourse. These analyses have covered, for example, work on suicide risk, Reddit-based mental health support groups, and psychiatric medication discourse (18–20). This work has established useful methodological precedents while also raising ethical concerns about inferring mental health states from social media data (21).

Within ADHD specifically, a growing body of literature has examined online discourse related to linguistic indicators of symptoms and emotional states (22), misinformation and stigma in ADHD-related TikTok content (23), public sentiment and perceptions of ADHD and autism spectrum disorder on X/Twitter (24), treatment and medication-related discourse (25), the quality of user-generated ADHD content (26), and online peer support and community identity (27–29). For example, Guntuku et al. (22) found that adults with self-reported ADHD on Twitter used more negative and self-critical language, with posts reflecting emotional dysregulation, substance use, and exhaustion. However, much of this work has focused on Twitter/X or TikTok, with comparatively less attention paid to how ADHD-related coping strategies are discussed on Reddit or how such discourse differs between women-focused and general ADHD online communities.

These gaps are especially relevant to women-focused ADHD communities, given that ADHD in women remains underrepresented in clinical research. Everyday online communities offer a naturalistic, complementary window into how ADHD-related symptoms and coping efforts are discussed in users’ own words. Reddit is well suited for this purpose because it provides semi-anonymous, peer-driven spaces that can facilitate self-disclosure and social support (28) while hosting both a women-focused ADHD community and a large general ADHD community. This enables a direct community-level comparison of ADHD-related discourse.

Based on the foregoing, the present study applies computational social science methods to examine linguistic indices of ADHD-related symptom expression and coping strategy within Reddit subcommunities, including r/ADHD, a central hub for ADHD-related discussion. Specifically, we compare how ADHD is discussed within a women-focused community (r/adhdwomen) versus a general ADHD community (r/ADHD) by analyzing patterns of symptom expression and coping strategies as articulated in everyday language. Through this formative analysis, we aim to inform the development of gender-sensitive diagnostic and intervention approaches grounded in real-world discourse and the lived contexts of adult ADHD.

## 2 Materials and methods

### 2.1 Data collection and preprocessing

We selected Reddit because its semi-anonymous, topic-segmented structure supports extended, self-disclosed narratives, which are ideal for capturing symptom and coping experiences in everyday language (19, 28, 29). Additionally, Reddit is distinctive in that it hosts both a dedicated women-focused ADHD community (r/adhdwomen) and a large general ADHD community (r/ADHD). This makes Reddit well suited for a direct community-level comparison of how ADHD is discussed by predominantly women and gender-diverse groups versus a broader general ADHD community.

We collected data from these two communities as follows. The community r/adhdwomen primarily targets women with ADHD, whereas r/ADHD includes users with ADHD more broadly. We used the hot, new, and top categories as of July 21, 2025, to gather a comprehensive dataset. Duplicate posts and irrelevant content were removed, and unnecessary spaces, hyperlinks, and special characters were eliminated during preprocessing. A total of 3,383 unique posts (1,414 from r/adhdwomen and 1,969 from r/ADHD) were prepared for analysis. According to its community rules, “In order to post or comment here, you must not identify primarily as a cisgender man,” r/adhdwomen intentionally excludes cisgender men. Given that Reddit does not provide demographic data, r/adhdwomen was assumed to reflect the experiences of users who identify as female or are gender diverse. We also assumed that the r/ADHD broadly included male, female, and gender-diverse individuals.

#### 2.1.1 Ethical considerations

This study is part of a two-year research project titled “Analyzing the Characteristics and Experiences of Women with ADHD through Text Mining and Qualitative Research” approved by the Institutional Review Board of the National Medical Center (IRB No. 2025-02-008). All analyzed data consisted of publicly available Reddit posts collected in accordance with the Reddit Terms of Service. No identifiable information was collected, and all data were anonymized before processing. In accordance with institutional and national ethics guidelines, the analysis was considered minimal risk and did not require additional participant consent.

#### 2.1.2 Text classification

GPT-assisted classification was used to support large-scale coding within a predefined coding framework, accessed through the official OpenAI Python API (model GPT-4.1-mini, version GPT-4.1-mini-2025-04-14). All analyses were conducted in Python 3.11, and the model temperature was fixed at 0.2 to minimize randomness in model responses and to increase consistency and reproducibility.

Two separate classification systems were developed to capture two dimensions (1): symptom expression and (2) coping strategies. Each system was informed by DSM-5 diagnostic criteria, existing ADHD-related literature reviews, and expert consultations, resulting in a customized JSON-based coding framework (criteria.json). Details on the classification of the symptom expression and coding of the coping strategies as well as on the postprocessing of the data can be found in the Supplementary Material. The model was constrained to assign only predefined category IDs from the coding schema and was not permitted to generate new analytic categories. Each post was coded as binary presence/absence for each subcategory; repeated mentions within the same subcategory were counted only once per post. Additional exploratory checks for uncategorized symptom-related expressions outside the primary schema are described in the Supplementary Material.

##### 2.1.2.1 Symptom expression classification

The symptom expression classification consisted of three main categories (A–C) and the following subcategories:

A. Core Symptoms (DSM-5)
A1: Inattention
A2: Hyperactivity and Impulsivity
B. Additional ADHD-Related Features
B1: Emotional Dysregulation and Internalizing Symptoms
B2: Social Difficulties and Camouflage
B3: Cognitive and Executive Function Deficits
B4: Risk Behaviors and Externalizing Symptoms
C. Comorbidities and Contextual Factors
C1: Comorbid Clinical Conditions (Developmental, Neuro, Psychiatric, or Somatic)
C2: Academic and Occupational Impairments
C3: Hormonal and Life Cycle Influences
C4: Diagnostic Delays/Misdiagnoses
C5: Gender Roles and Diagnostic Bias

Once symptom expressions were identified, the GPT model assigned one or more subcategory tags to each post and excerpts associated with each tag were stored as textual evidence. Of the 3,383 preprocessed posts (1,414 on r/adhdwomen and 1,969 on r/ADHD), 2,372 posts (927 [65.6%] on r/adhdwomen and 1,445 [73.4%] on r/ADHD) contained identifiable symptom expressions and were subsequently coded into one or more symptom expression categories. Frequencies and proportions by group were analyzed using chi-square tests and p-values ​​were adjusted using false discovery rate (FDR) correction.

##### 2.1.2.2 Coping strategy coding

The coping strategy classification consisted of four main categories (A–D) and the following subcategories:

A. Core Treatments
A1: Medication
A2: Psychotherapy (Cognitive behavioral therapy (CBT), Dialectical behavior therapy (DBT), counseling, psychoeducation)
B. Supportive Strategies
B1: Executive Function Support (time management, planning, organization, and professionally guided coaching)
B2: Mindfulness and Emotion Regulation (meditation, breathing, emotional awareness)
B3: Lifestyle (exercise, sleep, diet)
B4: Digital Tools and Technology Support (ADHD apps, productivity tools, AI tools such as ChatGPT)
B5: Social Support (family, friends, peer groups)
C. Environmental and Life Design
C1: Environmental Structuring (minimalism, routines, distraction control)
C2: Life and Career Design (career/educational choices, life redesign)
D. Other Strategies
D1: Other Strategies Not Included in A–C

Under a revised multilabel coding framework, all explicitly mentioned coping strategies within each post were retained and assigned to one or more predefined coping strategy subcategories. Thus, a single post could contribute to multiple coping strategy categories. Of all posts, 480 from r/adhdwomen (33.9%) and 751 from r/ADHD (38.1%) contained at least one explicitly coded coping strategy. The GPT model identified all individual strategies mentioned in each post and recorded their occurrence as binary indicators. Manual review of the extracted text confirmed that the retained strategies were explicitly described and contextually relevant (e.g., “start taking medication,” “ask your partner for help,” “set up phone alerts”).

#### 2.1.3 Human validation and output reproducibility

We evaluated GPT-assisted symptom expression classification using two human validation procedures. First, for excerpt-level labeling fidelity, two independent coders, both of whom are co-authors (S.R. and S.K.), reviewed 187 model-extracted evidence records and assigned symptom categories using the same coding schema. This step assessed whether model-surfaced evidence supported the assigned labels but did not assess missed content in the original posts.

Second, for full-text validation, two coders, both of whom are co-authors (S.R. and L.Z.), independently coded a separate stratified sample of 100 complete posts without access to the model output, including posts identified as symptom-negative by the model. Disagreements were adjudicated by the first author (J.S.) to create a human gold standard. Model performance was evaluated using precision, recall, F1 score, and Cohen’s κ, primarily at the consolidated 11-category level.

To assess reproducibility, 200 posts were reclassified three times under the same model and temperature settings for both symptom expression and coping strategy tasks. Run-to-run agreement was evaluated using exact label-set match, Jaccard overlap, Fleiss’ κ, and Krippendorff’s α.

### 2.2 Lexical and exploratory analysis of symptom expression

Lexical analysis was conducted on text segments containing the identified symptom expressions, not on all community posts. To examine linguistic trends specific to symptom-related discourse, word frequency and log-odds ratio (OR) analyses were used to compare term usage between r/adhdwomen and r/ADHD.

Word frequency distributions were visualized using WordCloud (a Python package), an exploratory tool for contextualizing key lexical themes. Log-OR analysis measured differences in lexical prominence by comparing the relative frequency of terms between the two communities, stabilizing estimates for low-frequency words while capturing relative lexical bias. Positive log ORs indicated more frequent use of that word in r/adhdwomen, whereas negative log ORs indicated greater prominence of the word in r/ADHD. These analyses were not used for statistical inference but served as a guide for subsequent quantitative comparisons.

### 2.3 Pairwise association analysis of coping strategies

To examine how coping strategies were combined within posts, we constructed subreddit-specific post-level binary matrices for all coping strategy subcategories identified under the revised multilabel coding framework. For each possible pair of coping strategy subcategories, we calculated co-occurrence counts, ORs, 95% confidence intervals (CIs), Fisher’s exact test *p*-values, and lift values. Lift was defined as the ratio of the observed co-occurrence frequency to the expected independent frequency of two strategies:

Lift(A, B) = P(A, B)/[P(A) × P(B)].

FDR correction was applied using the Benjamini–Hochberg procedure within each subreddit. To reduce unstable estimates driven by very rare combinations, interpretation in the main text focused on strategy pairs with at least five co-occurrences.

### 2.4 Heatmap visualization of pairwise associations

To facilitate comparison of coping structures between communities, significant positive associations were visualized as side-by-side heatmaps created for r/adhdwomen and r/ADHD. In these heatmaps, the cells represent significant coping strategy pairs after FDR correction and the color intensity reflects the magnitude of the association expressed as log_2_-transformed ORs.

## 3 Results

### 3.1 Differences in symptom expression

As shown in **Table 1**, percentages were calculated using only posts expressing ADHD-related symptoms (r/adhdwomen: n = 927; r/ADHD: n = 1,445) out of the total posts collected from each subreddit (N = 1,414 and N = 1,969, respectively). Group differences were tested using chi-square or Fisher’s exact tests with FDR correction. A statistically significant difference (FDR-adjusted p-value < 0.05) was observed for inattention (A1), which was more frequently reported in r/ADHD. By contrast, emotion dysregulation and internalizing symptoms (B1) were reported significantly more often in r/adhdwomen. Hormonal and life cycle influences (C3) were also mentioned significantly more frequently in r/adhdwomen. Other subcategories, including social difficulties and camouflage (B2), cognitive and executive function deficits (B3), risk behaviors and externalizing symptoms (B4), comorbid clinical conditions (C1), academic and occupational impairments (C2), delayed diagnosis and misdiagnosis (C4), and gender roles and diagnostic bias (C5), did not differ significantly between the two groups.

**Table 1:** Frequency of the ADHD-related symptom expression subcategories mentioned in r/adhdwomen and r/ADHD.

Subcategory	r/adhdwomen	r/ADHD	r/adhdwomen	r/ADHD	Test	FDR-adjusted	Significant
	n	n	(%, n = 927)	(%, n = 1445)		*p*-value	(FDR-adjusted *p*-value < 0.05)
A1. Inattention	639	1074	68.9%	74.3%	Chi-square	0.03	✅
A2. Hyperactivity and Impulsivity	95	152	10.2%	10.5%	Chi-square	0.89	ns
B1. Emotional Dysregulation and Internalizing Symptoms	491	683	53.0%	47.3%	Chi-square	0.025	✅
B2. Social Difficulties and Camouflage	57	71	6.1%	4.9%	Chi-square	0.43	ns
B3. Cognitive and Executive Function Deficits	208	338	22.4%	23.4%	Chi-square	0.80	ns
B4. Risk Behaviors and Externalizing Symptoms	31	30	3.3%	2.1%	Chi-square	0.21	ns
C1. Comorbid Clinical Conditions	52	74	5.6%	5.1%	Chi-square	0.30	ns
C2. Academic and Occupational Impairments	157	220	16.9%	15.2%	Chi-square	0.58	ns
C3. Hormonal and Life Cycle Influences	33	6	3.6%	0.4%	Chi-square	<0.001	✅
C4. Delayed Diagnosis and Misdiagnosis	19	26	2.0%	1.8%	Chi-square	0.86	ns
C5. Gender Roles and Diagnostic Bias	1	0	0.1%	0.0%	Fisher	0.60	ns

A word cloud was generated using only the symptom expression corpus (**Supplementary Figure 1**). In this visualization, word size reflected the relative frequency of lemmatized content words (nouns, verbs, adjectives, and proper nouns) after preprocessing and scaling. Both communities showed high frequency of core ADHD descriptors such as “work,” “focus,” “tasks,” and “forgetting,” reflecting difficulties with executive function and sustained attention. Posts in r/adhdwomen were lexically richer in emotional language, including terms such as “struggle,” “anxiety,” “overwhelmed,” and “want.

**Figure 1:**
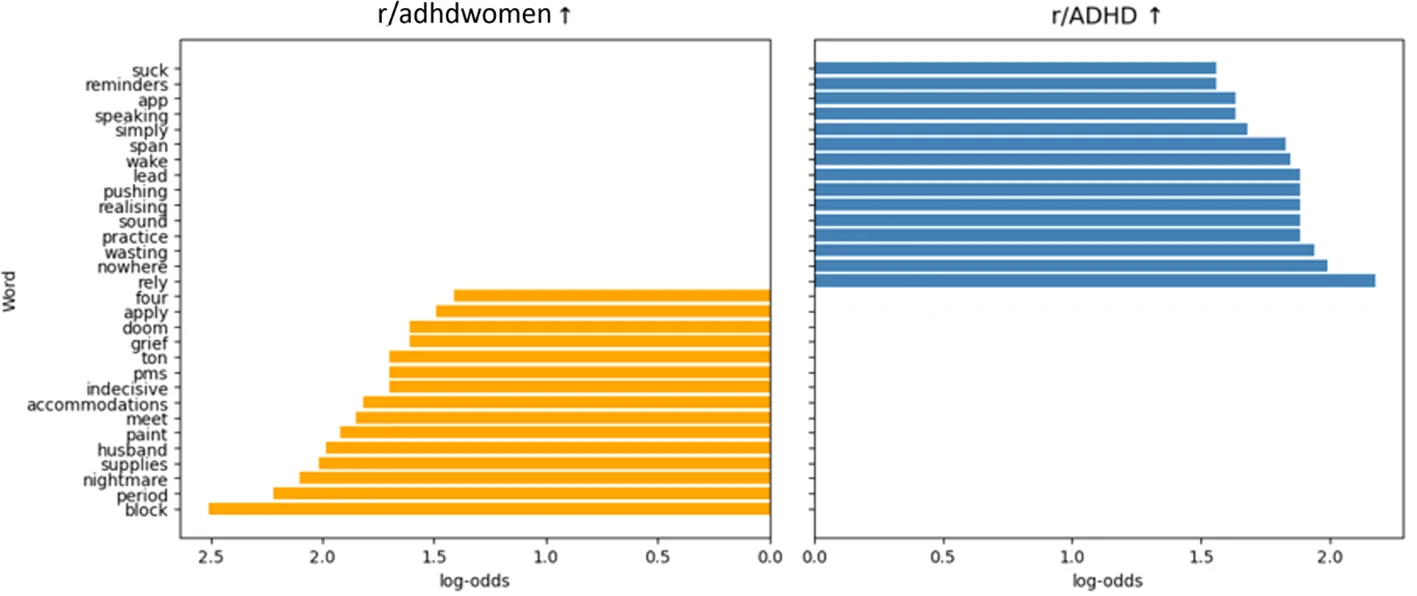
Top 15 differential words (log ORs) between r/adhdwomen and r/ADHD; Positive: r/adhdwomen bias (orange), Negative: r/ADHD bias (blue). The direction of the log ORs reflects relative lexical prominence rather than emotional polarity. Cells represent coping strategy pairs that remained significant after false discovery rate (FDR) correction; nonsignificant pairs are not displayed. Each cell shows the log₂ odds ratio (also encoded by color intensity, with higher values indicating stronger positive associations) and the FDR-adjusted p-value. Pairwise associations were calculated using Fisher’s exact test with FDR correction applied within each subreddit. Subcategory labels correspond to the coping strategy categories defined in the Methods section: A1 Medication, A2 Psychotherapy, B1 Executive Function Support, B2 Mindfulness and Emotion Regulation, B3 Lifestyle, B4 Digital Tools and Technology Support, B5 Social Support, C1 Environmental Structuring, C2 Life and Career Design, D1 Other Strategies.

Log-OR analysis revealed distinct lexical biases in ADHD-related discourse across the two Reddit communities (**Figure 1**). Words with positive log ORs (orange) were more frequent in r/adhdwomen, indicating greater reference to emotional (e.g., nightmare, indecisive, grief, doom), interpersonal (e.g., husband, accommodations), and hormonal experiences (e.g., period [menstruation], PMS). Conversely, words with negative log ORs (blue) were more common in r/ADHD, highlighting cognitive and attentional themes as well as task and time management topics (e.g., wasting, practice, sound, reminders, app, span, pushing). These results suggest a different linguistic framing of ADHD symptom expression, with r/adhdwomen tending to use more emotional and contextual language to describe symptoms.

### 3.2 Differences in coping strategies

As shown in **Table 2**, comparing the frequency of coping strategy subcategories mentioned in r/adhdwomen and r/ADHD revealed no statistically significant differences after FDR correction (FDR-adjusted *p* > 0.05). This indicates that the two communities share a similar overall distribution of coping-related topics. However, psychotherapy (A2) was mentioned relatively more frequently in r/adhdwomen (14.4%) than in r/ADHD (9.3%) and this difference approached statistical significance (*p* = 0.06). This pattern suggests a relatively greater emphasis on psychosocial interventions within the women-focused ADHD community, although the current data do not allow for a definitive conclusion.

**Table 2:** Frequency of the coping strategy subcategories across r/adhdwomen and r/ADHD.

Subcategory	r/adhdwomen	r/ADHD	r/adhdwomen	r/ADHD	FDR-adjusted	Significant
	n	n	(%, n = 480)	(%, n = 751)	*p*-value	(FDR-adjusted *p*-value < 0.05)
A1. Medication	338	561	70.4%	74.7%	0.24	ns
A2. Psychotherapy	69	70	14.4%	9.3%	0.06	ns
B1. Executive Function Support	164	220	34.2%	29.3%	0.24	ns
B2. Mindfulness and Emotion Regulation	35	73	7.3%	9.7%	0.30	ns
B3. Lifestyle	90	153	18.8%	20.4%	0.61	ns
B4. Digital Tools and Technology Support	43	63	9.0%	8.4%	0.81	ns
B5. Social Support	86	123	17.9%	16.4%	0.61	ns
C1. Environmental Structuring	55	74	11.5%	9.9%	0.61	ns
C2. Life and Career Design	5	16	1.0%	2.1%	0.30	ns
D1. Other Strategies Not Included in A–C	11	17	2.3%	2.3%	0.97	ns

Although the frequency of the coping strategy categories did not differ significantly between the two subreddits, pairwise association analysis identified several recurrent coping combinations within each community (**Table 3**). Across both subreddits, significant positive associations were consistently observed for B1 + C1 (executive function support + environmental structuring), B1 + B4 (executive function support + digital tools and technology support), and B1 + B5 (executive function support + social support), indicating that executive function-oriented coping was repeatedly discussed together with environmental, technological, and interpersonal support.

**Table 3:** Significant pairwise associations between the coping strategy subcategories within each subreddit.

Strategy pair	r/adhdwomen (co-occurrence n)	r/ADHD (co-occurrence n)	Lift (r/adhdwomen/r/ADHD)	OR (95% CI) (r/adhdwomen/r/ADHD)	FDR-adjusted *p*-value	Significant (FDR-adjusted *p*-value < 0.05)
B1 + C1	34	40	2.00/2.25	4.49 (2.48–8.11)/4.83 (2.88–8.08)	<0.001/<0.001	✅
B1 + B4	27	36	2.00/2.19	4.26 (2.22–8.18)/4.40 (2.59–7.47)	<0.001/<0.001	✅
B1 + B5	38	59	1.40/1.87	1.97 (1.22–3.18)/3.42 (2.29–5.13)	0.034/<0.001	✅
A2 + B5	24	–	1.94	3.00 (1.71–5.28)	0.002	✅
B2 + B3	14	–	2.16	3.29 (1.60–6.76)	0.013	✅
B3 + D1	–	8	2.44	3.87 (1.47–10.22)	0.036	✅

Subcategory labels correspond to the coping strategy categories defined in the Methods section: A1 Medication, A2 Psychotherapy, B1 Executive Function Support, B2 Mindfulness and Emotion Regulation, B3 Lifestyle, B4 Digital Tools and Technology Support, B5 Social Support, C1 Environmental Structuring, C2 Life and Career Design, D1 Other Strategies. FDR-adjusted p-values are shown as reported; values below 0.001 are denoted as < 0.001.

Several subgroup-specific associations also emerged. In r/adhdwomen, A2 + B5 (psychotherapy + social support) and B2 + B3 (mindfulness and emotion regulation + lifestyle) were also significant, suggesting recurring combinations that linked psychosocial and emotion regulation strategies. In r/ADHD, B3 + D1 (lifestyle + other strategies) was also significant. Overall, these findings suggest differences in recurring coping combinations across communities despite similar overall frequencies of individual coping strategy categories.

**Figure 2:**
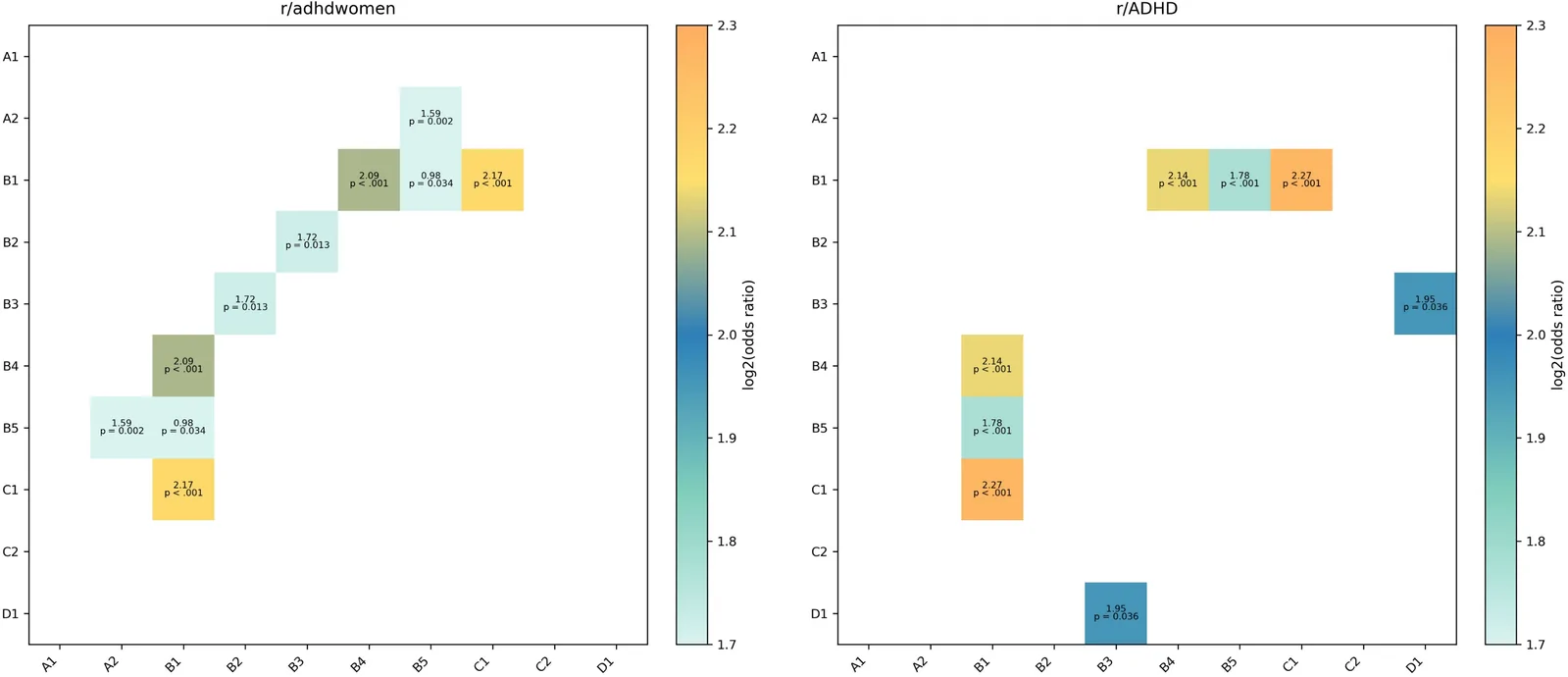
Heatmaps of the significant positive pairwise associations between the coping strategy subcategories in r/adhdwomen and r/ADHD.

**Figure 2** provides a side-by-side heatmap visualization of the significant positive pairwise associations within each subreddit. The heatmap reflects enriched pairwise associations after accounting for baseline prevalence rather than raw frequencies. Although medication (A1) was the most frequently mentioned coping strategy category overall, the pairwise association results indicate that the most clearly enriched combinations were more consistently observed around executive function support (B1). Given that medication was highly prevalent across posts in both communities, it likely functions as a broadly shared background component of coping discourse rather than as a selectively concentrated pairing strategy.

### 3.3 Human validation and output reproducibility

In the excerpt-level validation, human–human agreement was high (pooled Cohen’s κ = 0.94), whereas GPT–human agreement was moderate (κ ≈ 0.50; micro-F1 ≈ 0.58 at the detailed category level). In the full-text validation sample, human–human agreement was moderate (κ = 0.50). Against the adjudicated human gold standard, model performance at the primary 11-category level was also moderate, with precision = 0.67, recall = 0.56, F1 = 0.61, and κ = 0.53. Agreement was higher for emotional dysregulation/internalizing symptoms (F1 = 0.75) and hormonal/lifecycle influences (F1 = 0.82).

Run-to-run reproducibility was high. Exact label-set match across three runs was 81.0% for symptom expression and 93.0% for coping strategies, with Fleiss’ κ/Krippendorff’s α of 0.90 and 0.94, respectively. Full validation and reproducibility metrics are provided in Supplementary Material, Section 1.7.

## 4 Discussion

### 4.1 Differences in symptom expression

This study analyzed the verbal expression of ADHD symptoms in the Reddit communities r/ADHD and r/adhdwomen to examine community-level differences in perceived ADHD symptoms. Given that r/ADHD is a general, mixed-gender community rather than a male-only comparison group, all differences reported below should be interpreted as differences between online communities, not as differences between women and men with ADHD. Both groups described difficulties primarily within the DSM-5 core domains of inattention. Nevertheless, other associated difficulties of emotional dysregulation, executive dysfunction, and academic and occupational difficulties were frequently mentioned across communities and were more common than the DSM-5 core symptoms of hyperactivity/impulsivity. Further analysis showed that the linguistic composition and narrative framing of these symptoms differed in several ways.

Computational analyses of social media have been applied to online discussions about a range of mental health conditions, including depression, anxiety, suicide risk, ADHD, and premenstrual conditions. These analyses are not intended as a substitute for clinical diagnosis or epidemiology, but rather as a naturalistic window into how people describe distress, seek validation, and negotiate treatment (18, 19, 21, 24, 30, 31). This approach is particularly useful in areas in which clinical consensus is still emerging, such as emotional dysregulation, hormonal influences, life-course influences, and self-directed coping strategies in women with ADHD. Accordingly, the present study used Reddit discourse to identify community-level patterns rather than estimating clinical prevalence.

Inattention (A1) was the most frequently mentioned symptom category in both communities. It was mentioned slightly less often in r/adhdwomen than in r/ADHD (68.9% vs. 74.3%, FDR-adjusted *p* = 0.03). This pattern should not be interpreted as evidence that women experience less inattention. Rather, these results may suggest that users in r/adhdwomen describe a broader range of psychosocial difficulties beyond core symptoms, such as inattention, or that general ADHD communities such as r/ADHD may emphasize the most widely recognized symptom domains.

By contrast, emotional dysregulation and internalizing symptoms (B1) were significantly more frequent in r/adhdwomen than in r/ADHD (53.0% vs 47.3%, FDR-adjusted *p* = 0.025). Many users organized their narratives around emotional experiences, such as anxiety, depression, low self-esteem, and rejection sensitivity, with emotionally charged expressions particularly prominent. Some users described a sense of shame they found difficult to put into words or recurring rejection sensitivity sufficiently intense to disrupt their sleep. These findings align with previous research showing that women with ADHD experience elevated levels of internalized emotional distress (6, 11). They also align with computational analyses of ADHD discourse (22, 29) and, most directly, with a recent comparison of subreddits reporting that discussions about ADHD centered around women foregrounded emotional well-being, diagnosis, and seeking support, whereas general discussions about ADHD foregrounded medication/treatment and work or academic challenges (32). This study builds on previous research by quantifying the co-occurrence patterns of hormonal/lifecycle discourse and analyzing coping co-occurrence patterns coping.

A particularly notable feature of symptom expression by r/adhdwomen users was the discussion of hormonal cycle fluctuations. Approximately 3–4% of posts explicitly mentioned menstrual cycles, pregnancy and childbirth, or menopause as factors affecting attention, motivation, or medication efficacy. Users described decreased concentration and heightened emotional instability around menstruation, worsening cognitive function during the menopausal transition, and changes in medication effectiveness after childbirth. Some users, for example, framed these fluctuations in specific terms. They asked how to manage heightened emotional states during the luteal phase and wondered whether worsening focus signaled perimenopause. Reports of altered medication effectiveness during menstruation were especially common, suggesting that hormonal fluctuations and medication interactions may require adjustments to treatment plans. Although these accounts were anecdotal, they were recurrent. Many posts expressed relief that others shared these experiences but also highlighted a lack of information and support on this issue. These observations reflect emerging evidence on hormonal influences on ADHD symptom variability and stimulant response (7, 12), underscoring the need for research and clinical approaches that incorporate hormonal cycle-related challenges in women with ADHD. Recent work also emphasizes examining ADHD symptomatology across the female life course in relation to hormonal transitions, identifying menstrual cycles, pregnancy, and menopause as critical yet understudied phases (16).

Although the influence of hormones on ADHD is increasingly described in clinical and review literature (12, 13, 16), studies of ADHD on social media have rarely examined hormonal/lifecycle discourse directly. Adjacent Reddit evidence comes from a premenstrual dysphoric disorder (PMDD) peer-support study, in which ADHD-related subreddits were the third most common cross-community mental health activity among r/PMDD users (30). This study examines this intersection from the perspective of ADHD, demonstrating that concerns relating to menstruation, pregnancy/postpartum, menopause, and medication response emerged within ADHD discourse. These concerns were more prevalent in the women-focused community than in the general community (3.6% vs 0.4%); nevertheless, this reflects user discussion rather than verified hormonal status or PMDD diagnosis.

Some of these differences may also reflect what users feel comfortable raising, or perceive as relevant, in a women-focused versus a mixed-gender community rather than differences in underlying experience; for example, menstrual or hormonal concerns may be discussed more readily where the audience is assumed to share them. This is consistent with interpreting the results as community-level discourse patterns rather than measures of symptom prevalence.

In both communities, similar behaviors were noted, such as masking and compensatory strategies in which participants attempted to conceal ADHD-related difficulties to maintain social and occupational functioning. For example, some users described hiding their difficulties so effectively that others would never guess and the constant effort required to keep that mask in place. These patterns, which were quantitatively identified and illustrated in r/adhdwomen, often appeared in relation to expected roles within relationships or domestic contexts, including responsibilities that were assumed or resented. Even when exhausted, participants described “keeping it all together,” suggesting that sociocultural gender role expectations of competence and caregiving encouraged them to take on additional responsibilities to maintain stability and connection. This aligns with findings from earlier research indicating that women with ADHD often compensate for external demands by relying on substantial internal effort to maintain functioning (6, 8).

Quantitative text analysis of the symptom expression data revealed both shared characteristics and lexical differences. In the word cloud, both communities used terms such as “work,” “forgetting,” “focus,” and “tasks” to describe everyday symptoms associated with attentional difficulties. However, r/adhdwomen exhibited a higher frequency of emotional vocabulary, including “struggle,” “anxiety,” “want,” and “bad” as well as terms related to emotional, relational, and physiological experiences. By contrast, r/ADHD used cognitive and task-management language more frequently such as “reminders,” “practice,” and “apps,” suggesting a different functional framing of symptom experiences. This does not imply that affective dimensions are absent in the general ADHD community; rather, they appear to be less explicitly foregrounded in symptom narratives.

### 4.2 Differences in coping strategies and pairwise associations

In both communities, medication (A1) was the most frequently mentioned coping strategy, and executive function support (B1), lifestyle (B3), social support (B5), psychotherapy (A2), and environmental structuring (C1) were commonly mentioned as nonpharmacological approaches. For example, posts described using bullet journals, wall planners, and running to-do lists to externalize organization and track daily tasks. After FDR correction, the differences between the subgroups were not statistically significant, indicating that the distribution of coping strategy categories was generally similar across the two subreddits. This medication-centered, treatment-oriented emphasis is consistent with prior ADHD treatment-discourse research (25) and earlier subreddit comparisons (32).

Differences emerged more clearly in the pattern of pairwise associations than in the distributions of categories. Whereas prior ADHD social media studies have primarily focused on symptoms, sentiment, misinformation, and treatment topics, coping co-occurrence has received little attention. Its analysis suggests that between-community differences lie more in how strategies are combined than in their overall frequencies. Across both r/adhdwomen and r/ADHD, significant positive associations were consistently observed for B1 + C1 (executive function support + environmental structuring), B1 + B4 (executive function support + digital tools and technology support), and B1 + B5 (executive function support + social support). These recurring pairs suggest that executive function-oriented coping was often discussed together with environmental, technological, and interpersonal forms of support in both communities. Given that environmental structuring and digital tools share an organizational purpose with executive function support, the B1 + C1 and B1 + B4 pairs likely reflect a coherent cluster of organization-oriented strategies that users assemble together rather than associations among fully independent approaches.

Nevertheless, several subgroup-specific associations were observed. In r/adhdwomen, A2 + B5 (psychotherapy + social support) and B2 + B3 (mindfulness and emotion regulation + lifestyle) were additionally significant, indicating repeated pairings involving psychosocial and emotion regulation-oriented strategies. In r/ADHD, B3 + D1 (lifestyle + other strategies) also remained significant. In r/ADHD, the “other strategies” category included heterogeneous suggestions that did not clearly fit the predefined coping strategy categories (e.g., self-talk, supplement use such as 5-HTP, and practices like “ADHD tax” and planned break days). These research findings suggest that although the frequency of individual coping strategies does not differ significantly between the two communities, the way such strategies are combined shows some differences. These differences were clarified through heatmap visualization. Rather than simply reflecting raw frequencies, the heatmaps revealed strengthened pairwise associations after accounting for the baseline frequency rates. Therefore, although medication (A1) was the most frequently mentioned individual coping strategy category overall, the strongest strengthened pairwise associations were more consistently observed in relation to executive function support (B1).

Taken together, these results suggest that differences in coping discourse across subreddits may be better explained by recurring combinations of strategies than by simple differences in the frequency of individual categories. The additional combinations of psychotherapy + social support and mindfulness and emotion regulation + lifestyle observed in r/adhdwomen may show a slightly higher tendency for psychosocial and emotion regulation strategies to co-occur in women-centered communities. This aligns with accounts from online communities of adults with ADHD as sources of interpersonal connection and peer support (27, 33). It also suggests that, in the women-focused community, emotional validation can be discussed alongside more formal strategies. This may reflect a tendency toward such combinations of strategies for managing these symptoms. This finding links back to the observation that emotional dysregulation and internalizing symptoms were more prevalent in r/adhdwomen than in the other group in terms of symptom expression. However, since these coping strategies are based on an analysis of discourse patterns at the subreddit level rather than on direct evidence of treatment efficacy, they should be approached with caution.

From a clinical perspective, these results are consistent with the idea that managing ADHD involves a combination of pharmacological and nonpharmacological approaches rather than relying on one strategy. International clinical guidelines generally recommend a multimodal treatment approach combining pharmacotherapy with psychosocial and behavioral interventions such as psychoeducation, CBT, and environmental or organizational support (34, 35). This study suggests how these approaches are discussed and informally organized within online ADHD communities in everyday life.

Notably, the frequent combination of executive function support with environmental, technological, and interpersonal strategies reflects this multimodal logic, as users assemble it themselves outside of formal care settings. Interpreted in this way, the patterns are best understood as raising awareness rather than being prescriptive. The prominence of references to emotional dysregulation and internalizing symptoms alongside hormonal cycles in r/adhdwomen suggests that clinicians evaluating women with suspected or diagnosed ADHD may benefit from considering affective and hormonal factors that fall outside the DSM-5 core criteria. Recognizing that patients frequently raise such concerns in peer settings is also important.

### 4.3 Study limitations

This study is meaningful in that it explores the specific and realistic challenges faced and the coping strategies used through rich discourse data from online communities. However, several limitations should be acknowledged. Given that the sample was limited to Reddit users, participants were primarily Western and English-speaking. Therefore, generalizing the findings to broader populations beyond these online communities is difficult and potential cultural influences were not captured.

In addition, this study could not verify whether all users had received a formal clinical diagnosis, nor could it determine treatment status, comorbidities, or symptom severity. Such diagnostic ambiguity is a recognized limitation of social media-based mental health research in which analyses often rely on self-disclosed conditions and imperfect ground-truth labels rather than clinically validated diagnoses (36, 37). The data therefore reflect self-described accounts, which may introduce bias. Given that these characteristics cannot be confirmed, the observed discourse patterns cannot be mapped onto clinically validated phenotypes, and the clinical implications discussed above should be regarded as provisional. For example, the greater frequency of emotional and hormonal discourse in r/adhdwomen reflects what users chose to report rather than verified differences in severity, treatment response, or comorbidity burden. These findings are therefore best positioned as hypothesis-generating signals that warrant confirmation in clinically characterized samples before informing practice.

Given that Reddit does not host a community specifically for men with ADHD, this study compared a women-focused subreddit (r/adhdwomen) with a general ADHD subreddit (r/ADHD), which includes users of multiple gender identities. Therefore, r/adhdwomen may not represent the full range of female ADHD-related experiences on Reddit and the influence of gender within r/ADHD could not be evaluated. Accordingly, this study highlights community-level differences rather than definitive gender differences, limiting the interpretation of gender-specific effects.

Furthermore, GPT-assisted classification may not fully capture contextual meaning or intent. Human validation showed moderate GPT–human agreement and incomplete recall, indicating that some relevant expressions, especially secondary or context-dependent symptoms in multilabel posts, may have been missed. Therefore, category frequencies should be interpreted as approximate indicators of community-level discourse rather than exhaustive counts or prevalence estimates. Given that the same schema, prompt logic, and model settings were applied to both communities, this approach reduced procedural differences in coding across groups. However, category- or community-specific recall differences cannot be fully excluded. The findings should therefore be interpreted as exploratory discourse-level signals requiring confirmation in clinically characterized samples.

We also assessed run-to-run reproducibility for both symptom expression and coping strategy classification, and repeated model runs showed high output stability. However, reproducibility by itself does not establish validity. Given that human gold-standard validation focused on symptom expression categories, the coping strategy findings, particularly co-occurrence patterns, should be interpreted descriptively and warrant further validation in future work. Despite these efforts, the findings should be regarded as preliminary and hypothesis-generating.

The coping strategy categories are also not fully orthogonal—executive function support, environmental structuring, and digital tools share an organizational function (Section 4.2)—although the scheme additionally distinguishes modalities that target distinct mechanisms (medication, psychotherapy, lifestyle, mindfulness, and interpersonal support). Hence, executive function support represents one modality among several rather than a superordinate category.

Finally, this study did not include interaction-level indicators such as comment volume, likes, and posting times. Only the verbal content of posts was examined. Future research could explore the social role of online ADHD communities by analyzing interaction patterns and network structures to understand how discussions are shaped and reinforced. Nonetheless, this study highlights the potential of online communities to provide valuable supplementary information for understanding ADHD-related experiences and coping strategies beyond traditional clinical settings.

## 5 Conclusion

This study found that the women-focused ADHD community (r/adhdwomen) more frequently discussed emotional dysregulation and internalizing symptoms as well as hormonal and life cycle influences than the general ADHD community (r/ADHD). Within this community context, ADHD-related experiences were described not only in terms of attentional difficulties but also in broader emotional, interpersonal, and physiological terms. The two communities showed broadly similar overall frequencies of individual coping strategy categories, whereas they partly differed in the recurring combinations of strategies discussed within posts.

These findings suggest that women-focused ADHD discourse on Reddit may reflect dimensions of symptom experience and self-management that are not fully captured when ADHD is framed primarily in terms of core attentional and behavioral symptoms. Notably, in both communities, the DSM-5 core symptoms of hyperactivity/impulsivity were mentioned less frequently than other symptoms. However, the present results should be interpreted as community-level discourse patterns rather than direct evidence of gender-specific treatment use or treatment effectiveness.

Overall, this study highlights the value of social media text as a supplementary source for examining how ADHD is described and managed in everyday life. Future research could further validate these patterns in clinically characterized samples and examine how symptom expression and coping strategies vary across social, cultural, and clinical contexts.

## Data Availability

The data analyzed in this study were collected from publicly accessible posts in the subreddits r/adhdwomen and r/ADHD (https://www.reddit.com/r/adhdwomen/ and https://www.reddit.com/r/ADHD/). To protect user privacy and comply with platform terms, the raw post text is not redistributed. Derived data and analytic code are available from the corresponding author upon reasonable request.
