# Supplementary material for: Symptom expression and coping strategies in women-focused attention-deficit/hyperactivity disorder communities: a comparative natural language analysis of Reddit
Source: Front Psychiatry. 2026 Aug 7;17:1867179. doi: 10.3389/fpsyt.2026.1867179 (PMC13495778; doi:10.3389/fpsyt.2026.1867179)
Supplement: Supplementary file 1 [file Supplementaryfile1.docx]

Supplementary Material

# 1 Supplementary Data

## 1.1 Original Prompt Used for Automated Symptom Categorization

You are a clinical assistant AI. You will be given a short social media post written in free text.

Your task is to perform two types of analysis:

---

\### Part 1: ADHD Symptom Identification

Determine which ADHD-related symptom subcategories the text clearly and directly matches,

using the provided ADHD symptom schema (below).

- Only include subcategories that are **explicitly and clearly expressed** in the user’s text.

- Do **not** infer symptoms based on assumptions or indirect clues.

- For each matching subcategory, return:

- subcategory_id: the ID of the subcategory from the schema (e.g., "A1", "B2", "C3")

- evidence_text: a **list of direct quotes or phrases** from the user_text that clearly support this subcategory.

- Each `subcategory_id` must appear **only once** in the output.

If no subcategories apply, return an empty list.

---

\### Part 2: ADHD Coping Efforts

Determine whether the user mentions **efforts to cope with or overcome ADHD symptoms**.

If yes:

- Set `"mentioned"` to true.

- Extract and list the coping strategies/tools/actions mentioned (e.g., "used timer app", "took ADHD medication").

- Evaluate the user's description of the outcome using the following 3-point scale:

- "Very effective"

- "Somewhat effective"

- "Not effective"

If no such efforts are mentioned, set `"mentioned"` to false and omit the other fields.

---

\### Output Format

Return a JSON object in the following structure:

{

"symptoms": [

{

"subcategory_id": "<subcategory_id>",

"evidence_text": ["<excerpt1>", "<excerpt2>", ...]

},

...

],

"coping_efforts": {

"mentioned": true | false,

"strategies": ["<description1>", "<description2>", ...], // only if mentioned == true

"effectiveness": "Very effective" | "Somewhat effective" | "Not effective" // only if mentioned == true

}

}

---

\### ADHD Symptom Schema

{

"A": {

"name": "Core Diagnostic Symptoms (DSM-5 Based)",

"subcategories": {

"A1": {

"name": "Inattention",

"items": [

"Careless mistakes",

"Difficulty sustaining attention",

"Doesn’t listen",

"Doesn’t follow through",

"Difficulty organizing",

"Avoids sustained mental effort",

"Loses things",

"Easily distracted",

"Forgetful"

]

},

"A2": {

"name": "Hyperactivity / Impulsivity",

"items": [

"Fidgeting or squirming",

"Leaves seat",

"Inappropriate running/climbing or restlessness",

"Noisy play",

"On the go behavior",

"Talks excessively",

"Blurts out answers",

"Difficulty waiting turn",

"Interrupts/intrudes on others"

]

}

}

},

"B": {

"name": "Clinical Presentations and Symptom Expressions",

"subcategories": {

"B1": {

"name": "Emotional Dysregulation and Reactivity",

"items": [

"Emotional dysregulation",

"Emotional lability",

"Rejection sensitivity",

"Alexithymia"

]

},

"B2": {

"name": "Mood and Internalizing Symptoms",

"items": [

"Anxiety",

"Depression",

"Shame/guilt",

"Demoralization",

"Internalizing distress"

]

},

"B3": {

"name": "Social Functioning Difficulties",

"items": [

"Peer rejection",

"Social withdrawal",

"Relational aggression",

"Verbal aggressiveness",

"Reduced prosocial behaviors"

]

},

"B4": {

"name": "Social Camouflaging and Compensation",

"items": [

"Masking/Camouflaging",

"Overcompensation",

"Performing as a false self",

"Scaffolding (external structuring)"

]

},

"B5": {

"name": "Cognitive and Executive Functioning Deficits",

"items": [

"Executive dysfunction",

"Mind wandering",

"Forgetfulness/lateness",

"Subtle processing difficulties"

]

},

"B6": {

"name": "Impulsivity and Risky Behaviors",

"items": [

"Risky sexual behavior",

"Teenage pregnancy",

"Substance use/self-medication",

"Gambling/shopping",

"Self-harm",

"Suicidal ideation or attempts"

]

}

}

},

"C": {

"name": "Comorbidities and Modulating Factors",

"subcategories": {

"C1": {

"name": "Developmental and Neuropsychological Comorbidities",

"items": [

"Autism spectrum traits",

"Language disorder",

"Dyslexia",

"Learning disabilities/difficulties"

]

},

"C2": {

"name": "Psychiatric and Somatic Comorbidities",

"items": [

"Eating disorders",

"Sleep difficulties",

"Chronic fatigue",

"Pain syndromes",

"Fibromyalgia"

]

},

"C3": {

"name": "Academic and Occupational Impairments",

"items": [

"Academic underachievement",

"Work-related impairment",

"Poor time management"

]

},

"C4": {

"name": "Hormonal and Lifecycle Influences",

"items": [

"Menstrual symptom worsening",

"Pregnancy-related variability",

"Postpartum worsening",

"Menopausal worsening",

"Estrogen-dopamine interaction",

"Puberty-related shifts",

"Thyroid dysfunction"

]

},

"C5": {

"name": "Delayed Diagnosis and Misdiagnosis",

"items": [

"Delayed diagnosis",

"Misdiagnosis as mood or personality disorder",

"Subthreshold or late-onset presentations"

]

},

"C6": {

"name": "Gender Role Expectations and Diagnostic Bias",

"items": [

"Gendered behavioral expectations",

"DSM criteria male bias",

"Underrecognition by teachers or caregivers"

]

}

}

}

}

## 1.2 Post-processing Category Consolidation Procedure

Initial automated categorization of ADHD symptoms was performed using the symptom representation subcategory structure defined in the original prompt (A1–A2, B1–B6, C1–C6).

After obtaining the model’s raw output, conceptual overlap across several items was identified. Following expert methodological recommendations, semantically related subcategories were consolidated into broader thematic categories to reduce overlap.

This consolidation was not a rerun of the analysis using a revised prompt but a conceptual integration step applied to the model-generated labels.

The final analysis categories reported in the manuscript reflect these combined themes.

## 1.3 Mapping Between Original Subcategories and Final Category Structure

The table below summarizes the mapping between the original fine-grained subcategories used by the model and the final merged categories reported in the manuscript.

| Original Subcategory (Detailed Schema) | Final Category (Merged Structure) |
| --- | --- |
| B1. Emotional Dysregulation and Reactivity | B1. Emotional Dysregulation and Internalizing Symptoms |
| B2. Mood and Internalizing Symptoms | B1. Emotional Dysregulation and Internalizing Symptoms |
| B3. Social Functioning Difficulties | B2. Social Difficulties and Camouflage |
| B4. Social Camouflaging and Compensation | B2. Social Difficulties and Camouflage |
| B5. Cognitive and Executive Functioning Deficits | B3. Cognitive and Executive Function Deficits |
| B6. Impulsivity and Risky Behaviors | B4. Risk Behaviors and Externalizing Symptoms |
| C1. Developmental and Neuropsychological Comorbidities | C1. Comorbid Clinical Conditions |
| C2. Psychiatric and Somatic Comorbidities | C1. Comorbid Clinical Conditions |
| C3. Academic and Occupational Impairments | C2. Academic and Occupational Impairments |
| C4. Hormonal and Lifecycle Influences | C3. Hormonal and Life Cycle Influences |
| C5. Delayed Diagnosis and Misdiagnosis | C4. Diagnostic Delays / Misdiagnoses |
| C6. Gender Role Expectations and Diagnostic Bias | C5. Gender Role Expectations and Diagnostic Bias |

## 1.4 Exploratory Schema-Coverage Check for Uncategorized Symptom-Related Expressions

As an exploratory schema-coverage check, the first author reviewed posts not assigned to the predefined symptom categories to determine whether they contained broader ADHD-related difficulties outside the primary analytic schema. These expressions were summarized as uncategorized symptom-related content and were not retained as primary analytic categories because they were heterogeneous and did not yield interpretable category-level comparisons. This exploratory review was used to contextualize schema coverage, not to generate additional quantitative symptom categories.

## 1.5 Original Prompt Used for Automated Coping Strategies Categorization

You are an assistant who analyzes user input to identify ADHD coping strategies used by the user, based on a

predefined 2-level category system.

Your task is to return **ALL distinct strategies mentioned** in the text, never just one. Each detected strategy

must be represented as a separate JSON object inside an array.

- Do not merge multiple strategies into one object.

- Do not omit any strategy that is explicitly described.

---

\### Category System

A. Core Treatments
 - A1: Medication
 - A2: Psychotherapy (CBT, DBT, counseling, psychoeducation)

B. Supportive Strategies

- B1: Executive Function Supports (time management, planners, coaching, task breakdown, environment

organization)
 - B2: Mindfulness & Emotion Regulation (meditation, breathing, emotional awareness, impulse control)
 - B3: Lifestyle Habits (exercise, sleep, diet)
 - B4: Digital Tools & Tech Aids (ADHD apps, productivity tools, gamified tools, AI tools like ChatGPT)
 - B5: Social Support (help from family, friends, peer groups)

C. Environment & Life Design

- C1: Environment Structuring (minimalism, routine, distraction control)

- C2: Life & Career Design (career/education choices, life redesign)

D. Other Strategies
 - D1: Other strategies not fitting A–C

---

\### For each detected strategy, output:
- **subcategory_id** (e.g., "B1")
- **evidence_text**: array of quotes from the text supporting the classification
- **effectiveness**: one of `"effective"`, `"not effective"`, or `"unknown"`

---

\### Strict Output Rules:

1. Always return a **valid JSON array**.

2. Each strategy must be a **separate JSON object** in the array.

3. If multiple strategies appear in the text, the JSON array must contain multiple objects.

4. If no strategies are clearly mentioned, return `[]`.

5. Do not include explanations, comments, or text outside of the JSON.

---

\### Example Output:

[

{

"subcategory_id": "B1",
 "evidence_text": [
 "I've started using a daily planner and it helps me stay on track.",
 "Breaking down tasks into smaller parts made things less overwhelming."

],

"effectiveness": "effective"

},

{

"subcategory_id": "A1",
 "evidence_text": [
 "I tried medication but it made me anxious and I had to stop."

],

"effectiveness": "not effective"

},

{

"subcategory_id": "B3",
 "evidence_text": [
 "Going for a morning run makes me more focused during the day."

],

"effectiveness": "effective"

}

]

## 1.6 Explanation of Prompt Fields Used in the Final Analysis

Although the original prompt included optional effectiveness rating items (“effective,” “not effective,” “unknown”), the quantitative analysis used only categorical identification of coping strategies.

In the initial analysis, effectiveness items were automatically extracted, but they were excluded because the qualitative descriptions of effectiveness were highly variable and did not allow for reliable comparison. Therefore, effectiveness assessments were not included in the quantitative results.

## 1.7 Validation and Reproducibility of GPT-Assisted Classification

### 1.7.1 Human Validation of Symptom Expression Classification

Human validation focused on symptom expression classification. In the first stage, two independent coders reviewed model-extracted evidence spans from 187 coded instances and judged whether each assigned category was supported by the text. Inter-rater reliability was high (pooled Cohen’s κ = 0.94), whereas the model agreement with the human consensus was moderate (κ = 0.50; micro-F1 = 0.58).

In the second stage, a fresh stratified sample of 100 posts, disjoint from the first validation sample and including model-negative posts, was coded in full text by two coders unaware of the model output. The sample included a random backbone (n = 70) and a rare-category top-up (n = 30). Disagreements were adjudicated without referencing the model output to create a human gold standard. Against this gold standard, model performance at the primary 11-category level was precision = 0.67, recall = 0.56, F1 = 0.61, and κ = 0.53. Human inter-rater agreement on the same full-text task was κ = 0.50. Recall estimated on the random backbone was 0.52. Per-category performance is shown in Supplementary Table 1.

**Supplementary Table 1**. Per-category validation performance against the human gold standard for symptom expression classification

| Code | Symptom category | n (gold) | Precision | Recall | F1 |
| --- | --- | --- | --- | --- | --- |
| A1 | Inattention | 30 | 0.51 | 0.80 | 0.62 |
| A2 | Hyperactivity/Impulsivity | 11 | 0.62 | 0.45 | 0.53 |
| B1 | Emotional Dysregulation/Internalizing | 44 | 0.79 | 0.70 | 0.75 |
| B2 | Social Difficulties/Camouflage | 19 | 0.78 | 0.37 | 0.50 |
| B3 | Cognitive/Executive Deficits | 22 | 0.60 | 0.41 | 0.49 |
| B4 | Risk/Externalizing | 4 | 0.50 | 0.75 | 0.60 |
| C1 | Comorbid Clinical Conditions | 28 | 0.86 | 0.43 | 0.57 |
| C2 | Academic/Occupational | 15 | 0.50 | 0.53 | 0.52 |
| C3 | Hormonal/Lifecycle | 9 | 0.88 | 0.78 | 0.82 |
| C4 | Diagnostic Delay/Misdiagnosis | 16 | 1.00 | 0.38 | 0.55 |
| C5 | Gender Role/Bias | 3 | — | 0.00 | — |
|  | Overall (micro-averaged) | 201 | 0.67 | 0.56 | 0.61 |

**Note.** Metrics were calculated against the adjudicated human gold standard for the full-text validation sample. Because posts could receive multiple labels, the total number of gold-standard labels exceeds the number of posts. Precision and F1 were not estimable for C5 because no model-predicted positive labels were available for that category. Category-level metrics for low-frequency categories should be interpreted cautiously. The overall row reports micro-averaged performance across the 11 symptom categories.

### 1.7.2 Run-to-Run Reproducibility Analysis

To evaluate output stability, 200 randomly selected posts from both subreddits were reclassified three times per task under the original settings (GPT-4.1-mini-2025-04-14, temperature = 0.2), yielding 1,200 API calls. All calls returned a valid JSON object. Treating the three runs as replicate raters, exact label-set match across all three runs was 81.0% for symptom expression and 93.0% for coping strategies. Mean pairwise Jaccard overlap was 0.922 and 0.975, respectively. Any-label versus none agreement was 95.5% and 98.5%, respectively, and overall post-by-label cell agreement was 97.8% and 99.1%, respectively. Fleiss’ κ / Krippendorff’s α was 0.897 for symptom expression and 0.942 for coping strategies (Supplementary Table 2).

Per-subcategory reliability was lowest for low-prevalence or broadly defined categories, such as academic/occupational impairment (κ = 0.65) and “other” strategies (κ = 0.71), and highest for high-prevalence categories, such as inattention (κ = 0.90) and medication (κ = 0.98).

**Supplementary Table 2**. Run-to-run reproducibility of GPT-assisted classification

| Metric | Symptom expression | Coping strategies |
| --- | --- | --- |
| Posts analyzed | 200 | 200 |
| Repeated runs per post | 3 | 3 |
| Exact label-set match across three runs | 81.0% | 93.0% |
| Mean pairwise Jaccard overlap | 0.922 | 0.975 |
| Any-label vs. none agreement | 95.5% | 98.5% |
| Overall post-by-label cell agreement | 97.8% | 99.1% |
| Fleiss’ κ | 0.897 | 0.942 |
| Krippendorff’s α | 0.897 | 0.942 |
| Mean labels assigned per run | 1.09 | 0.58 |

**Note.** A random sample of 200 posts spanning both subreddits was reclassified three times for each task under the original settings, GPT-4.1-mini-2025-04-14 with temperature = 0.2. The analysis comprised 1,200 API calls, all of which returned valid JSON. Fleiss’ κ and Krippendorff’s α were calculated over post-by-subcategory binary presence/absence cells. These metrics quantify run-to-run reproducibility, not agreement with the human gold standard.

# 2 Supplementary Figure

**Supplementary Figure 1.** Symptom expression word clouds derived from r/adhdwomen and r/ADHD posts

r/adhdwomen_Symptom Expression (final filtered, common scale)

r/ADHD_Symptom Expression (final filtered, common scale)
